# Oxidative Status in Adult Anorexia Nervosa Patients and Healthy Controls—Results from a Cross-Sectional Pilot Study

**DOI:** 10.3390/antiox11050842

**Published:** 2022-04-26

**Authors:** Jolana Wagner-Skacel, Fiona Haidacher, Markus Wiener, Karoline Pahsini, Sabine Marinschek, Theresa Lahousen, Willibald Wonisch, Susanne Bengesser, Mary I. Butler, Sonja Lackner, Andreas Meinitzer, Dietmar Enko, Sabrina Mörkl

**Affiliations:** 1 Department of Psychiatry and Psychotherapeutic Medicine, Medical University of Graz, 8036 Graz, Austria; jolana.wagner-skacel@medunigraz.at (J.W.-S.); fiona.haidacher@stud.medunigraz.at (F.H.); markus.wiener@stud.medunigraz.at (M.W.); karoline.pahsini@medunigraz.at (K.P.); sabine.marinschek@medunigraz.at (S.M.); theresa.lahousen@medunigraz.at (T.L.); susanne.bengesser@medunigraz.at (S.B.); 2 Department of Medical Psychology and Psychotherapy, Medical University of Graz, 8036 Graz, Austria; 3 Division of Physiological Chemistry, Medical University of Graz, 8036 Graz, Austria; willibald.wonisch@medunigraz.at; 4 Department of Psychiatry and Neurobehavioral Science, University College Cork, T12 YT20 Cork, Ireland; mary.butler@ucc.ie; 5 Division of Immunology and Pathophysiology, Otto Loewi Research Center, Medical University of Graz, 8036 Graz, Austria; sonja.lackner@medunigraz.at; 6 Division of Medical and Clinical Laboratory Diagnostics, Medical University Graz, 8036 Graz, Austria; andreas.meinitzer@medunigraz.at (A.M.); dietmar.enko@medunigraz.at (D.E.)

**Keywords:** anorexia nervosa, oxidative stress, total oxidative capacity, total antioxidative capacity, endogenous peroxidase, antibodies against oxidized LDL, polyphenols, TMAO

## Abstract

Oxidative stress describes an imbalance of reactive oxygen species (ROS) and antioxidative defence systems. Recently, the consequences of oxidative stress have become a central field of research and have been linked to the genesis of multiple psychiatric diseases. Some oxidative stress parameters have not been investigated before in anorexia nervosa (AN) patients, including the gut microbiota-derived metabolite trimethylamine N-oxide (TMAO) and polyphenols (PPm). In this cross-sectional pilot study, we evaluated these markers together with total peroxides (TOC), antioxidative capacity (TAC), endogenous peroxidase activity (EPA) and antibodies against oxidized LDL (oLAb) in serum samples of 20 patients with AN compared to 20 healthy controls. The antioxidative capacity was significantly decreased in AN patients, with a mean TAC of 1.57 mmol/L (SD: ±0.62); t (34) = −2.181, *p* = 0.036) compared to HC (mean = 1.91 mmol/L (SD: ±0.56), while the other investigated parameters were not significantly different between the two groups. In AN patients, TAC correlated with EPA (r_sp_ = −0.630, *p* = 0.009). This study suggests that there is an antioxidative deficiency in AN patients. In this respect, there is a demand for interventional studies to determine whether antioxidants can be used as add-on therapy in the treatment of AN.

## 1. Introduction

Anorexia nervosa (AN) is a severe mental disorder characterized by an intense fear of weight gain and a disrupted body image with weight loss behaviours [1]. AN has a high relapse rate [2], with mortality rates of around 5–6% in the initial years, increasing up to 20% in chronic cases [2]. Metabolic dysregulation, energy imbalance and homeostatic disruption play an essential role in AN. Neuroendocrine, gastrointestinal, immunological and cardiovascular disorders contribute to the premature death of AN patients [3]. Difficulties in weight gain are attributed to a lack of compliance and/or resulting from changes in metabolism, e.g., deficiencies in the ability to extract energy from food. The body fat mass serves as protection for organs and storage of energy as well as endocrine tissue. Adipose tissue, responsible for the production of cytokines and the hormones leptin and adiponectin, has a pathophysiological impact on the course of disease, with lower levels of leptin and higher levels of adiponectin in patients with AN [4].

There have only been few studies evaluating the oxidative status in patients with AN. Some previous studies have shown that patients with AN exhibit alterations in the oxidative state, with imbalances between the production of free radicals (e.g., reactive oxygen species (ROS); reactive nitrogen species (RNS)) and antioxidants [5,6]. This is caused by the overproduction of free radicals or insufficient antioxidant defence [7]. Oxidative stress may damage biomolecules, impair cell structures and negatively impact organ functions, leading to the activation of transcription factors and resulting in inflammation [8]. Oxidative stress is also associated with various mental disorders which are comorbidities of AN, such as depression [9]. Interestingly, inflammatory processes in depressive patients can frequently result in increased reactive oxygen and nitrogen species’ production [10,11].

Screening methods, such as the biomarkers total antioxidant capacity (TAC), total oxidant capacity (TOC), antibodies against oxidized low-density lipoprotein (oLAb), and endogenous peroxidase activity (EPA), are widely used to determine oxidative stress and antioxidant capacity [12,13].

Additionally, the level of nutritional antioxidants such as polyphenols in the circulation can be used to assess the antioxidant potential of the patient’s diet. In particular, plant-based diets such as the Mediterranean diet are known for their antioxidative effects [14]. They are characterized by high consumption of fruits and vegetables, which is associated with a higher intake of polyphenols [14,15]. Polyphenol microtitre (PPm) determines the total amount of polyphenols in serum samples [16]. In accordance with the patient’s habitual diet, the concentrations of antioxidants in plasma may be elevated and thus so might TAC levels [14].

Peroxides are one of the first lipid peroxidation breakdown products. This biomarker indicates oxidative stress in individuals [17,18]. Antibodies against oLAb can be viewed as a second line of defence against oxidative stress, and have been shown to represent long-term oxidative stress [19]. Endogenous peroxidase activity is a part of the body’s enzymatic antioxidative defence system. The gut microbiota-derived metabolite trimethylamine N-oxide (TMAO) has been shown to promote oxidative stress and inflammation, contributing to various diseases (e.g., atherosclerosis, cardiovascular disease, chronic kidney disease, inflammatory bowel disease, neurodegenerative disease) [20].

To our knowledge, TMAO and PPm have never been investigated in AN patients before, and oLAb as well as EPA were only investigated in one previous study by our group [6]. Identifying novel biomarkers that are capable of predicting the course of the disease or symptom severity would help develop therapies to prevent relapse. We conducted a single-centre pilot study to investigate oxidative stress markers and antioxidant capacity along with parameters of eating pathology in individuals with AN. We hypothesized that (1) oxidative stress markers would be significantly higher in patients with AN than in the healthy control group, and that (2) oxidative stress parameters would correlate with depression and eating disorder severity scores.

## 2. Materials and Methods

### 2.1. Participants

In this study, a total of 40 female subjects were enrolled, comprising 20 AN patients and 20 control (HC) subjects. AN patients were recruited at the inpatient psychiatry ward at the department for Psychiatry and Psychotherapeutic Medicine, Medical University of Graz, Austria between November 2017 and January 2021. All AN patients received treatment as usual pharmacotherapy. For the AN group, a diagnosis of the disorder using the DSM IV criteria for AN had to be met. Exclusion criteria included neurological or psychiatric diseases apart from AN, head injuries, trauma, drug or alcohol abuse, nicotine abuse and acute suicidality. HCs were recruited from the personal environment of the hospital and administrative staff. Demographical and clinical data were collected. Body Mass Index (BMI) was calculated as weight (kg)/height in (m^2^). All participants could withdraw from the study at any moment without providing any justification. All participants gave written informed consent and were interviewed on their medical history. This study was approved by the Ethics Committee of the Medical University Graz (approval number EK 26-383 ex 13/14) and was conducted according to the Declaration of Helsinki.

### 2.2. Blood Sampling

Fasting venous blood samples were drawn from an antecubital vein from the seated subject as part of a routine laboratory checkup. The blood samples were centrifuged and immediately frozen at −20 °C for intermediate storage and then transferred to allow further storage at −80 °C and investigations of TOC, PPm, EPA, TAC and oLAb.

### 2.3. Oxidative Stress Biomarkers

Peroxides (TOC) and the endogenous peroxidase activity (EPA) were determined with rapid enzymatic in vitro diagnostic assays by Labor Diagnostic Nord (LDN, Nordhorn, Germany) [18]. The assay system is based on a peroxide/peroxidase reaction using 3,5,3′,5′-tetramethybenzidine (TMB) as substrate. Results were calculated from a linear standard curve and peroxide levels were presented as µmol H_2_O_2_ equivalents, while peroxidase activity was presented as U/L [21].

Total polyphenols were determined with a commercially available kit from Omnignostica Ltd. (Omnignostica Ltd., Höflein/D., Austria). This method is based on the reaction of polyphenols with transition metals in the presence of the Folin–Ciocalteu reagent. The optical density of the dark-colored complex was measured at a wavelength of 766 nm. Serial dilutions of gallic acid were used to determine the standard curve [22].

A colourimetric method by Labor Diagnostic Nord (Nordhorn, Germany) was used to determine TAC. TAC is used to measure the antioxidative capacity against the attack of prooxidative radicals, among others, in body fluids such as serum and plasma. TMB was used as the substrate to measure the colour change induced by radicals, initiated through a peroxide/peroxidase reaction. Antioxidants in the sample quench radicals, and thus inhibit the reaction of ROS with TMB, which is associated with an indirect proportional signal. After pipetting the reagents and samples into the microtiter plate, they were incubated for 20 min at 4 °C. After that, 50 µL stop solution was added, and the samples were measured at a wavelength of 450/620 nm (microplate reader: Power Wavex Bio-Tek Instruments Inc., Santa Clara, CA, USA) [16]. Results were presented as mmol/L.

A commercial enzyme immunoassay (ELISA) supplied by Biomedica (Biomedica, Vienna, Austria, catalogue no: BI-20032) was used to determine the titres of antibodies against oxidized low-density lipoprotein (oLAb). The ELISA is based on the binding reaction of the prediluted samples to the previously oxidized LDL by cupric ions and bound to the microtiter wells. Detection was performed by the binding of a secondary, peroxidase-coupled anti-IgG antibody which permitted colorimetric detection with TMB as substrate. Results are expressed as mU/mL [21].

TMAO was analysed with a stable isotope dilution assay and high-performance liquid chromatography (HPLC) on a SCIEX QTRAP 4500 triple-quadrupole instrument (Applied Biosystems, Framingham, MA, USA) equipped with an Agilent 1260 Infinity HPLC system (Agilent Technologies, Santa Clara, CA, USA) [23].

### 2.4. Psychological and Dietary Scales

We used the Eating Disorder Examination Questionnaire (EDEQ) [24], a self-administered questionnaire containing 28 questions, to characterize eating disorder pathology. The EDEQ contains four sub-scales: restraint, shape concern, eating concern, and weight concern. As an index of AN symptoms, the short version of the EAT questionnaire with 26 items (EAT-26) was used [25].

To rule out cognitive deficits, the mini-mental state examination (MMSE) [26] was used. The Beck Depression Inventory (BDI) [27] and Hamilton Depression Rating Scale (HAMD) [28] were used to grade the severity of depressive symptoms. The Mediterranean Diet Score (MDS) is a questionnaire conducted to see how closely individuals adhere to a Mediterranean diet. Higher scores indicate a closer adherence [29].

### 2.5. Statistical Analyses

All analyses were performed using IBM SPSS Statistics 27.0 (IBM Corp, Armonk, NY, USA). The Kolmogorov–Smirnov test was used to test for normal distribution. Frequencies, means/median values, and standard deviations/interquartile range were calculated to describe the population. Group differences were tested either with nonparametric tests (Mann–Whitney U Test) or independent samples t-test if normal distribution was present. Depending on the characteristics of variables, correlations of parameters were calculated using Spearman’s correlations (r_sp_) or Pearson’s correlations (r). *p*-values below 0.05 were considered statistically significant. Missing values from questionnaires and oxidative stress parameters were excluded from the analysis. The total number of participants required for the study was estimated according to G Power analysis using G*Power 3.1.9.4 [30] software (Universität Kiel, Kiel, Germany, source: https://www.psychologie.hhu.de/arbeitsgruppen/allgemeine-psychologie-und-arbeitspsychologie/gpower.html (accessed on 21 April 2022)) using data from a cross-sectional study on TMAO [31], to 15 persons per group (noncentrality parameter δ = 3.86; critical t = 2.04; df = 28, actual power= 0.96).

## 3. Results

### 3.1. Group Characteristics

Table 1 shows the demographical and anthropometric data of study participants. Height, weight and BMI were normally distributed, while age was not normally distributed. The subjects of both groups were matched for age. As was expected, weight and BMI between the AN and HC groups showed statistically significant differences. Out of the patient cohort, one patient took selective serotonin reuptake inhibitors (SSRI) and four took other antidepressants at the time of sampling. One patient had a combination therapy of SSRI and an antipsychotic, and another patient took other antidepressants and an antipsychotic. In total, 13 patients were medication-free. No participant in the control cohort took any psychopharmacological medication. One of 20 patients had the purging type of AN, all other participants had the restrictive type.

### 3.2. Psychological Evaluation and Questionnaires

Table 2 shows the scores of psychological tests and questionnaires regarding eating pathology and depression in all participants. The EDEQ subscale “shape concern” delivered normally distributed scores, while the other scales were not normally distributed. Significant differences between both groups were witnessed in the total scores of EDEQ, as well as all subscores. Furthermore, AN patients showed considerably higher results in both depression rating scales (HAMD and BDI).

### 3.3. Oxidative Stress Biomarkers

**Figure 1:**
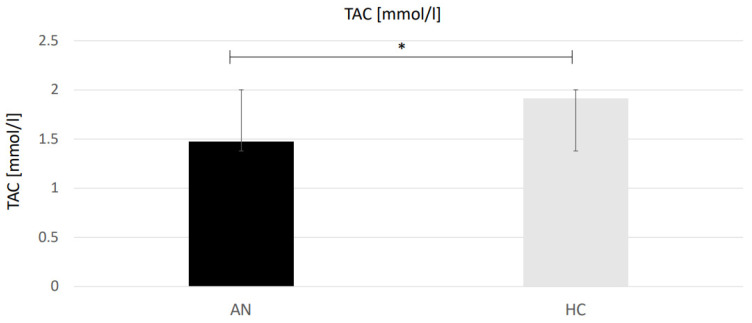
Differences in the antioxidative capacity between anorexia nervosa patients and controls. AN = anorexia nervosa, HC = healthy controls, TAC = total antioxidant capacity. * = significant difference, *p* < 0.05.

The biomarkers TMAO, TAC and PPm were normally distributed, while TOC, oLAb and EPA did not show normal distribution. TAC was the only biomarker that showed a significant difference between groups (see Table 3, Figure 1). TAC was significantly decreased in the AN group (M = 1.47, SD = 0.62) compared to the HC group (M = 1.91, SD = 0.56, t(34) = −2.181, *p* = 0.036). Serum polyphenols, peroxidase activity, peroxides and antibodies against oxidized LDL did not show significant differences between the AN and HC group.

### 3.4. Correlations

Correlations were calculated between parameters of oxidative stress, scores of depressive symptoms (BDI, HAMD), scores of eating pathology (EDEQ total) and Mediterranean diet (MDS) separately for both groups.

In the HC group, TOC correlated with PPm (r_sp_ = 0.482, *p* = 0.031), with BMI (r_sp_ = 0.739, *p* < 0.001) and weight (r_sp_ = 0.459, *p* = 0.048). TAC correlated significantly with EPA (r_sp_ = −0.470, *p* = 0.036) and EAT-26 score (r_sp_ = −0.574, *p* = 0.020). EPA correlated significantly with weight (r_sp_ = 0.505, *p* = 0.027). EDEQ total showed no significant correlation with any oxidative stress parameter but correlated significantly with BDI (r_sp_ = 0.577, *p* = 0.019) and EAT-26 score (r_sp_ = 0.522, *p* = 0.038). HAMD scores correlated significantly with EAT-26 score (r_sp_ = 0.561, *p* = 0.024l).

**Figure 2:**
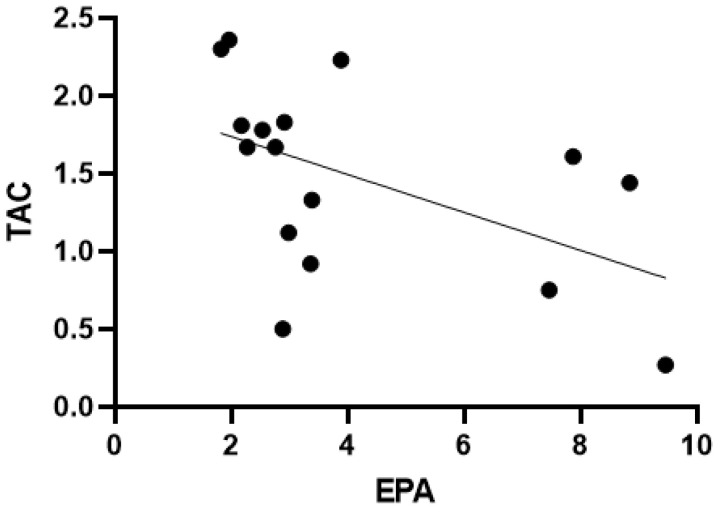
Correlation of TAC and EPA in AN patients. AN = anorexia nervosa, TAC = total antioxidant capacity, EPA = endogenous peroxidase activity.

In the AN group, TOC correlated with TAC (r = −0.607, *p* = 0.013) and height (r_sp_ = −0.572, *p* = 0.016). TAC correlated with EPA (r_sp_ = −0.630, *p* = 0.009) (see Figure 2) and MDS (r = 0.644, *p* = 0.013). EDEQ total showed significant correlations with HAMD (r = 0.599, *p* = 0.011), and EAT-26 score (r = 0.846, *p* = <0.001). EAT-26 score correlated with BMI (r = 0.694, *p* = 0.003) and weight (r = 0.755, *p* = 0.001). TMAO correlated with TAC (r = 0.573, *p* = 0.032).

## 4. Discussion

In this pilot study, we investigated, for the first time, TMAO and PPm in AN patients compared to healthy controls, along with oLAb and EPA (which were only investigated in one previous study by our group [6]), TAC and TOC. We found that AN patients showed a significantly lower TAC, while the other parameters were not significantly different from controls.

As was expected [6], we found a significantly lower TAC in AN patients compared to healthy controls. A large number of factors, including BMI, are associated with a higher TAC. In a previous study, TAC increased after achieving a healthy BMI in 25 patients with AN [18]. Therefore, TAC could potentially represent a valid biomarker of nutritional status in AN patients [18,32]. Our findings are also consistent with those seen in a recent study on 111 female patients with AN that found a 43% decrease in plasma TAC in the AN cohort compared to HC. This study described an inverse correlation between the duration of disease and this biomarker [33]. In an interventional study, TAC increased significantly after patients reached a healthy BMI [32].

The reduced levels of TAC in AN patients may indicate a lower availability of antioxidants. This may be attributed to either a low intake of fruits and vegetables or a higher turnover of antioxidants due to increased oxidant processes. A relatively high intake of fruits and vegetables in AN patients compared to healthy controls has been observed, and elevated levels of plasma carotenoids as a marker of fruits and vegetables have been detected [34], however, in our study, polyphenols were not significantly different between AN patients and HC. Additionally, the bioavailability of fat-soluble nutrients may be disturbed in AN patients, which may potentially lead to reduced TAC levels.

In patients with other psychiatric disorders, decreased TAC has also been reported. A study comparing the dietary and serum TAC levels in 30 healthy and 30 depressed male university students showed significantly lower serum TAC in the depressed group. Low dietary intake of antioxidative substances, such as vitamin C and carotenoids, is likely the main contributor [35]. Comparable results have been reported on a cohort of 57 patients with major depression (46 of them female) contrasted with a HC group of 40 adults. After antidepressant treatment, however, TOC dropped significantly and TAC rose significantly within the depression group [36]. The cause–effect relationship between TAC and AN should be a question for future clinical studies to answer the question of whether low food intake is responsible for the reduced TAC or whether an antioxidant deficiency causes it. In this respect, antioxidative supplementation in AN patients should be therapeutically investigated.

While TAC was significantly decreased in AN patients in comparison to the HC group, neither TMAO, PPm, EPA, TOC, or oLAb were found to be significantly different. These findings indicate that TOC may be influenced by factors other than nutrition, or TAC neutralized elevated TOC. While TAC seems to be affected in underweight and malnourished patients, TOC could be more connected to high amounts of body fat [16,21,37,38]. For example, previous research has shown an increase in TOC in obese children compared to a group of non-obese pre-adolescents, with a positive correlation between the duration of the condition and the TOC-levels [39].

To our knowledge, this is the first study to measure TMAO as a biomarker in AN patients. Due to the frequently found gut dysbiosis in AN patients [40], we expected distinctly altered TMAO levels in AN patients compared to healthy controls, however, we could not confirm our hypothesis. Although TMAO is regarded as a gut-derived biomarker of cardiovascular risk, and although cardiovascular complications and arteriosclerosis are frequently described in AN patients [41,42], other mechanisms besides a rise in TMAO may be responsible.

The oLAb marker showed no significant differences between the AN and HC group. A possible cause could be the influence of antidepressant therapy discussed in the limitations. Lackner et al. assessed relative body weight and body fat in AN patients, and showed significantly lower antioxidative oLAb levels compared to reference values in patients with higher subcutaneous adipose tissue [6].

TAC correlated with EPA, as was previously also shown by Zelzer et al. [11], and was associated with adherence to the Mediterranean diet in AN-patients, as shown in [11]. Since the Mediterranean diet is high in foods containing antioxidants [15], these findings may indicate higher antioxidative capacity through increased ingestion of antioxidant-containing foods. AN patients show high rates of food addiction to specific, especially healthy, food, which correlates with psychopathology [43].

In AN patients, eating pathology correlated with depression measured with HAMD. The self-rating instrument BDI showed fewer symptoms than the diagnostic questionnaire used by psychiatrists (HAMD). This might be a consequence of impairments in the ability of self-perception and personality functioning.

We found statistically significant negative correlations between the measured TOC, BMI and weight in the HC group. As mentioned above, TOC may be connected to the amount of body fat, while TAC is more connected to the quality of nutritional intake.

### Limitations

One limitation of this study is the low sample size of 20 participants per group. However, most studies concerning AN patients have been performed with sample sizes ranging from 4–20 participants [44]. To our knowledge, this is the largest study yet conducted on AN patients which included a variety of antioxidative and oxidative stress biomarkers, some of which have not been measured before in these patients.

Additionally, this study focused on female patients; it would be interesting to investigate these parameters in male individuals in the future to see whether sex influences the results. Nonetheless, most patients with AN are female, making up 90% of cases [45]. The use of medications may have influenced the oxidative stress levels of some members of the AN group, as 7 out of 20 patients received psychopharmacological medication at time of testing. Some antidepressants, such as SSRI, have been shown to decrease oxidative stress in rat studies [46]. However, our obtained results did not change when patients with medication were excluded from the analyses. Further, we did not conduct nutritional interviews with patients to find potential nutritional patterns connected to oxidative stress status. Oxidative stress may differ between patients with the purging type and restrictive type of AN. However, only one patient in our sample had the purging type, therefore additional analyses of differences between the two subtypes were not possible. 

Despite these limitations, this study shows that oxidative stress in patients with AN is an interesting field that warrants further research with studies evaluating antioxidant intake in contrast to controls. To our knowledge, this is the largest study yet conducted in AN patients, which included a variety of oxidative stress biomarkers. Similar studies with larger sample sizes would be worthwhile.

## 5. Conclusions

Patients with AN had significantly decreased TAC compared to healthy controls, but not in other parameters such as TMAO and PPm. Oxidative stress parameters correlate with scores of eating pathology and depression. Interventional studies are needed to determine whether patients with AN could benefit from antioxidative strategies, such as special anti-oxidative supplements, sip food, Mediterranean style diet and perhaps the avoidance of certain medications known to cause oxidative stress, such as TCA, as an adjunct to usual treatment options [47]. Increasing patients’ caloric intake and focusing on individualized dietary approaches containing sufficient antioxidants to balance oxidative stress should be part of dietary rehabilitation. Specific meal plans including foods rich in Vitamins C, E, trace elements such as selenium and polyphenols could be created by nutritional experts and applied in a multidisciplinary treatment approach. However, evidence is still not strong enough for making clear recommendations in this area.

## Figures and Tables

**Table 1:** Anthropometric data; AN = anorexia nervosa, HC = healthy controls, M = mean, SD = standard deviation, Min = minimum, Max = maximum, IQR = interquartile rank.

	AN M (SD)/M (Min; Max; IQR)	HC M (SD)/M (Min; Max; IQR)	*p*-Value
Height [m]	1.64 (0.06)	1.68 (0.07)	0.007
Weight [kg]	37.03 (6.78)	64.32 (12.79)	<0.001
BMI [kg/m^2^]	13.71 (1.97)	22.23 (3.37)	<0.001
Age [years]	23 (18; 54; 20–30)	26 (22.00; 27; 25–30.75)	0.136

**Table 2:** Results of psychological questionnaires; AN = anorexia nervosa, HC = healthy controls, M = mean, SD = standard deviation, Min = minimum, Max = maximum, IQR = interquartile rank, EDEQ = Eating Disorder Examination Questionnaire, EAT-26 = Eating Attitude Test-26, MDS = Mediterranean Diet Score, HAMD = Hamilton Depression Rating Scale, BDI = Beck Depression Inventory.

Name of Questionnaire	AN M (SD)/M (Min; Max; IQR)	HC M (SD)/M (Min; Max; IQR)	*p*-Value
EDEQ shape concern	4.03 (1.43)	0.69 (0.66)	<0.001
EDEQ total	3.1 (0.76; 5.7; 1.54–4.47)	0.37 (0; 57; 0.23–0.71)	<0.001
EDEQ restraint	3.4 (0; 6; 0.25–4.95)	0.3 (0; 22; 0–0.85)	<0.001
EDEQ eating concern	4 (0; 5.2; 1.3–4.4)	0 (0; 1.2; 0–0.2)	<0.001
EDEQ weight concern	2.7 (1.6; 6; 2.2–4)	2 (0; 2.2; 0–1)	<0.001
EAT-26	26 (6; 51; 18.75–36.75)	2 (0; 8; 0–5.5)	<0.001
MDS	7 (2; 11; 3.75–9)	8 (5; 12; 7–9)	0.266
HAMD	18 (4; 31; 11–18)	1 (0; 8; 0–1)	<0.001
BDI	25.5 (1; 44; 13.5–33.5)	1 (0; 17; 0–1)	0.001

**Table 3:** Oxidative stress biomarkers. AN = anorexia nervosa, HC = healthy controls, M = mean, SD = standard deviation, Min = minimum, Max = maximum, IQR = interquartile rank, TAC = total antioxidant capacity, PPm = Polyphenols microtitre, TOC = total oxidative capacity, oLAb = Antibodies against oxidized low-density lipoproteins, EPA = endogenous peroxidase activity, TMAO = trimethylamine N-oxide.

Ox Stress Parameters	AN M (SD)/M (Min; Max; IQR)	HC M (SD)/M (Min; Max; IQR)	*p*-Value
TAC [mmol/L]	1.47 (0.62)	1.91 (0.56)	0.036
Ppm [mmol/L]	7.79 (0.52)	7.7 (0.34)	0.476
TOC [µmol/L]	138 (23; 355; 96–171)	144 (85; 573; 131–204.5)	0.427
oLAb [mU/mL]	513 (185; 6000; 185–2729.5)	513 (185; 6000; 366.5–1811.5)	0.408
EPA [U/L]	2.98 (1.82; 10.42; 2.40–7.67)	3.07 (1.11; 12.92; 2.55–7.53)	0.547
TMAO [µmol/L]	2.16 (1.01)	2.14 (1.05)	0.949

## Data Availability

Data are contained within the article and Supplementary Dataset S1.

## Supplementary Materials

The following supporting information can be downloaded at: https://www.mdpi.com/article/10.3390/antiox11050842/s1. Supplementary Dataset S1: Original data.
